# Prophylaxis of caries with fluoride for children under five years

**DOI:** 10.1186/s12887-021-02702-3

**Published:** 2021-09-08

**Authors:** Sophie Jullien

**Affiliations:** Barcelona Institute for Global Health, University of Barcelona, Barcelona, Spain

**Keywords:** Dental health, Dental caries, Fluorides, Prevention

## Abstract

We looked at existing recommendations and supporting evidence on the effectiveness and potential harms of the different fluoride interventions in preventing dental caries in children under 5 years of age.

We conducted a literature search up to the 12th of September 2019 by using key terms and manual search in selected sources. We summarized the recommendations and the strength of the recommendation when and as reported by the authors. We summarized the main findings of systematic reviews with the certainty of the evidence as reported.

Water fluoridation has been widely implemented worldwide for several decades and evidence shows it reduces the prevalence of dental caries. Salt or milk fluoridation are other collective fluoride interventions that are also effective to prevent dental caries in children. The evidence of effects of oral fluoride supplements for caries prevention is limited and inconsistent. The use of fluoride toothpastes has consistently been proven to be effective in the prevention of dental caries. The evidence for the effects of the different levels of fluoride concentration in toothpastes is more limited. Topical fluorides (gels and varnishes) are effective in preventing dental caries and are mainly recommended to children with high risk of dental caries. Early childhood intake of fluoride supplements and fluoride level of 0.7 ppm (ppm) in drinking water are associated with the risk of dental fluorosis, ranging from minor forms to severe forms that are of aesthetic concerns.

## Background

### Introduction

The World Health Organization (WHO) European Region is developing a new pocket book for primary health care for children and adolescents in Europe. This article is part of a series of reviews, which aim to summarize the existing recommendations and the most recent evidence on preventive interventions applied to children under 5 years of age to inform the WHO editorial group to make recommendations for health promotion in primary health care. In this article, we looked at existing recommendations and supporting evidence on the effectiveness and potential harms of the different fluoride interventions in preventing dental caries in children under 5 years of age.

### Why is fluoride important?

Fluoride has an important role in preventing caries, by acting in several ways. It increases dental mineralization and bone density; it has bactericidal action on cariogenic bacteria; and it delays demineralization and promotes enamel remineralization when present in dental plaque and saliva.

### Context

Dental caries is the most frequent oral disease worldwide. WHO estimated that in Europe, between 20 and 90% of children aged 6 years have dental caries [1]. Caries in children are a source of pain and can lead to loss of teeth, impaired growth and failure to thrive, and can affect speech, appearance, self-esteem and school performance [2]. Across Europe, caries is particularly prevalent among adults and children with low socioeconomic status. At the same time, those populations are disregarded or underserved by dental care in many countries due to inequity in access to dental care [3].

In the European region, the WHO estimated a decline in the average number of teeth affected by dental caries among 12 year-old children, from 3.0 in 1990 to 1.8 in 2015 [1]. Since the late 1960s, WHO has officially endorsed the use of fluoride for population-based prevention of dental caries. Fluoride has been recognised as the main factor responsible for the decrease of caries prevalence in the past decades. Universal access to fluoride for preventing caries was declared to be part of the basic right to human health during the WHO World Health Assembly in 2007 [4]. There are several methods of fluoride intervention used for the prophylaxis of caries in children. Fluoride can be delivered by systemic or topical administration. For systemic administration, there are collective (fluoridated water, milk and salt) and individual (fluoride oral supplements) delivery methods, and for topical use, fluoride can be professionally administered (fluoride gels and varnishes) or self-administered (toothpastes, and mouth-rinses for older children).

In this document, we review the effectiveness of these different fluoride interventions for preventing dental caries in children. Although assessed separately, it is however important to remember that combinations of different fluoride interventions are being used in different settings around the world [4].

### Terminology and acronyms

In this document, we will use the decayed-missing-filled (dmf) index, which is one of the most common methods for assessing the prevalence of dental caries. This index is used for dental caries affecting teeth (dmft for decayed, missing and filled teeth) or surfaces (dmfs for decayed, missing and filled surfaces). Written in lowercase letters (dmft or dmfs), it refers to the primary dentition.

## Key questions

1. How effective are the different fluoride interventions in preventing dental caries in children under 5 years of age?
2. What are the potential harms of fluoride interventions for preventing caries in children under 5 years of age?

For each fluoride intervention, we will also report effects of timing and dosages when described in the studies selected for replying question 1.

We provide a list of identified risk factors for caries in children.

## Search methods and selected manuscripts

We described the search methods, data collection and data synthesis in the second paper of this supplement (Jullien, S., Huss, G. & Weigel, R. Supporting recommendations for childhood preventive interventions for primary health care: elaboration of evidence synthesis and lessons learnt (2021) BMC Pediatrics. https://doi.org/10.1186/s12887-021-02638-8).

The search was conducted on the 12th of September 2019, by manual search and by using the search term “dental caries” and “fluoride”. We found several documents from the WHO, including a systematic review on the effects and safety of fluoride toothpastes, and reports on the Global Consultation on Public Health Intervention against Early Childhood Caries that was conducted recently. We included recommendations and their supporting evidence from the US Preventive Services Task Force (USPSTF) (2013–14), the PrevInfad workgroup from the Spanish Association of Primary Care Pediatrics (2011), and the American Academy of Pediatrics (AAP) (2014). The recommendations from the Centers of Disease Control and Prevention (CDC) date from 2001, and we therefore did not include them in this summary document [5]. We included a public health guideline from the National Institute for Health and Care Excellence (NICE) and an evidence-based toolkit for prevention of oral health published by Public Health England and endorsed by NHS.

The search in the Cochrane library returned 67 reviews and 11 protocols. By screening the titles and abstracts, we included seven systematic reviews and none of the protocols. We excluded the Cochrane review conducted in 2016 by Marinho et al. on fluoride mouth rinses for preventing dental caries in children and adolescents, as children included in this review were older than 5 years of age (due to the nature of the intervention) [6]. We also excluded the review that assessed the effectiveness of slow-release fluoride devices for the control of dental decay, as we considered this to be beyond the scope of the fluoride interventions to review in this document [7]. We identified one protocol on salt fluoridation for preventing dental caries but this protocol was withdrawn from publication (as out of date and not meeting current Cochrane methodological standards) [8].

All the included manuscripts for revision in this article are displayed in Table 1.

**Table 1:** Included manuscripts for revision

Sources	Final selected manuscripts
**WHO**	• Wright 2014 – Fluoride toothpaste efficacy and safety in children younger than 6 years. A systematic review [9] • O’Mullane 2016 – Fluoride and oral health (Evidence based approach, review and recommendations) [4] • Petersen 2016 – Prevention of dental caries through the use of fluoride – the WHO approach. Editorial [10] • WHO 2017 – Expert consultation on Public Health Intervention against Early Childhood caries [11] • Phantumvanit 2018 - WHO [12]
**USPSTF**	• Moyer 2014 – Recommendations [2] • Chou 2013 – Evidence support and systematic review [13]; full systematic review [14] • Community Preventive Services Task Force 2013 – Systematic review on Community Water Fluoridation [15]
**PrevInfad**	• 2011 recommendations and supporting evidence [16]
**AAP**	• Fluoride use in caries prevention in the primary care setting (recommendations) [17]
**NICE**	• Public health guideline on ‘Oral health: local authorities and partners’ 2014 [18] • Delivering better oral health: an evidence-based toolkit for prevention 2017 (NHS guidance linked to NICE) [19]
**Cochrane Library**	• Iheozor-Ejiofor 2015 – Water fluoridation for the prevention of dental caries (Systematic review) [20] • Yeung 2015 – Fluoridated milk for preventing dental caries (Systematic review) [21] • Walsh 2019 – Fluoride toothpastes of different concentrations for preventing dental caries (Systematic review) [22] • Tubert-Jeannin 2011 – Fluoride supplements (tablets, drops, lozenges or chewing gums) for preventing dental caries in children (Systematic review) [23] • Marinho 2013 – Fluoride varnishes for preventing dental caries in children and adolescents (Systematic review) [24] • Marinho 2015 – Fluoride gels for preventing dental caries in children and adolescents (Systematic review) [25] • Wong 2010 – Topical fluorides as a cause of dental fluorosis in children (Systematic review) [26]

*Abbreviations*: *AAP* American Academy of Pediatrics, *NICE* National Institute for Health and Care Excellence, *PrevInfad* PrevInfad workgroup from the Spanish Association of Primary Care Pediatrics, *USPSTF* US Preventive Services Task Force, *WHO* World Health Organization

## Existing recommendations

We summarize the existing recommendations and the strength of recommendations as per their authors in Table 2.

**Table 2:** Summary of existing recommendations

	Ref	Date	General recommendations for prophylaxis of caries with fluoride in children under five years	
			Systemic (collective or individual) fluoride	Topical fluoride
**WHO**	[4] [10] [11]	2016 2017	“When feasible, access to national fluoridation schemes using water, salt and milk as vehicles should be promoted.” “Community ***water fluoridation*** is safe and cost-effective and should be introduced and maintained wherever socially acceptable and feasible. The optimum fluoride concentration will normally be within the range 0.5–1.0 mg/L.” “***Salt fluoridation*** should be considered where water fluoridation is not feasible for technical, financial or sociocultural reasons. It can be used for small groups or large populations, is very economical and, where necessary, provides freedom of choice.”	“Affordable and effective fluoride toothpaste should be available for all children. Policy-makers and dental professionals should advocate for, and promote, legislation conducive to the affordability, accessibility and quality of fluoride toothpaste. Strategies should include the elimination of taxes on fluoride toothpaste and designation of the toothpaste as a health product and not a cosmetic product.” “Daily tooth brushing with fluoride toothpaste from the eruption of the first tooth must be regarded as the best clinical practice today” (moderate evidence). “Fluoride varnish can, to some extent, decrease caries incidence in early childhood” (low evidence). “Silver diamine fluoride can arrest dentine caries in primary teeth and prevent recurrence after treatment” (very low evidence).
	[12]	2018	“Confirm the use of community fluoride administration, such as **water, salt or milk** as primary prevention of early childhood caries”	“Perform toothbrushing for children by a parent twice daily, using a soft toothbrush of age-appropriate size.” “Use standard fluoride-containing **toothpaste** (1000–1500 ppm) in all children under the age of 6”
**USPSTF**	[2]	2014	For children ≤5 years: “**Oral fluoride supplementation** starting at age 6 months for children whose water supply is deficient in fluoride.” (B recommendation)	For children ≤5 years: “Apply fluoride **varnish** to the primary teeth of all infants and children starting at the age of primary tooth eruption.” (B recommendation)
**PrevInfad**	[16]	2011	**Fluoride supplements** Only for children with risk factors for developing dental caries and depending on the fluoride to drinking water: oral fluoride supplement from 6 months of age.	For children aged between 0 and < 2 years: tooth brushing is recommended with toothpaste containing 1000 ppm of fluoride in a quantity of ‘craping’ or ‘stain’. For children aged between 2 and 6 years: tooth brushing is recommended with toothpaste containing between 1000 and 1450 ppm of fluoride in a quantity of a pea.
**AAP**	[17]	2014	**Community water fluoridation:** Recommended for both groups at low and high caries risk. **Dietary fluoride supplements:** Recommended if drinking water supply is not fluoridated for both groups at low and high caries risk.	**Toothpaste:** recommended to start at tooth emergence (smear of paste until age 3 years, then pea-sized) for both groups at low ang high caries risk. **Fluoride varnish:** recommended every 3–6 months starting at tooth emergence, recommended for both groups at low and high caries risk. **Over-the-counter mouth rinse:** not applicable for groups at low caries risk; recommended to start at age 6 years if the child can reliable swish and spit in groups at high caries risk.
**NICE**	[18]	2014	Not addressed	“Ensure all early years services provide oral health information and advice”, including the “use of fluoride toothpaste as soon as teeth come through” “Consider fluoride varnish programmes for nurseries and primary schools in areas where children are at high risk of poor oral health”
**NHS**	[19]	2017	Not addressed	In children aged 0 to 3 years^a^: “As soon as teeth erupt in the mouth brush them twice daily with a fluoridated toothpaste” (Strength of the evidence I) “Brush last thing at night and on one other occasion.” (Strength of the evidence III) “Use fluoridated toothpaste containing no less than 1000 ppm fluoride.” (Strength of the evidence I) “It is good practice to use only a smear of toothpaste” (Strength of the evidence GP). In children aged 3 to 6 years: “Brush at least twice daily, with a fluoridated toothpaste” (Strength of the evidence I) “Brush last thing at night and at least on one other occasion.” (Strength of the evidence III) “Use fluoridated toothpaste containing more than 1000 ppm fluoride.” (Strength of the evidence I) “It is good practice to use only a pea size amount” (Strength of the evidence GP) “Spit out after brushing and do not rinse, to maintain fluoride concentration levels” (Strength of the evidence III) “Apply fluoride varnish to teeth two times a year (2.2% NaF-)” (Strength of the evidence I). For children aged 0 to 6 years giving concern: all advice as above plus: “Use fluoridated toothpaste containing 1350–1500 ppm fluoride.” (Strength of the evidence I) “Apply fluoride varnish to teeth two or more times a year (2.2% NaF-)” (Strength of the evidence I)

*Abbreviations*: *AAP* American Academy of Pediatrics, *NHS* National Health Service (UK), *NICE* National Institute for Health and Care Excellence, *ppm* parts per million, *PrevInfad* PrevInfad workgroup from the Spanish Association of Primary Care Pediatrics, *USPSTF* US Preventive Services Task Force, *WHO* World Health Organization

^a^The strength of the evidence was classified as follows [19]

I: Strong evidence from at least one systematic review of multiple well-designed randomized control trial/s

II: Strong evidence from at least one properly designed randomized control trial of appropriate size

III: Evidence from well-designed trials without randomization, single group pre-post, cohort, time series of matched case-control studies

IV: Evidence from well-designed non-experimental studies from more than one centre or research group

V: Opinions of respected authorities, based on clinical evidence, descriptive studies or reports of expert committees

GP for ‘Good practice’: specific evidence is not available but statements make practical sense

## Existing evidence

A series of documents and systematic reviews looked at the evidence of fluoride for preventing dental caries. ‘Fluoride and Oral Health’ is a comprehensive document that reports the supporting evidence of use of fluorides to improve dental and oral health, and as such provides background information and data for each fluoride intervention, but also considers cost and implementation of interventions [4]. The USPSTF commissioned a systematic review of the evidence on prevention of dental caries by primary care clinicians in children under 5 years of age, in order to update their 2004 recommendations [13]. This USPSTF review focused on three main areas: the effectiveness of various interventions for preventing caries, risk assessment for future caries, and screening for caries (beyond the scope of this summary). We report below the evidence from these documents, together with the findings from the seven Cochrane systematic reviews we included and the Wright 2014 review (see Table 1). Due to the extensity of the topic, we strived to report most recent data when we found data for the same topic from different sources.

## Efficacy of fluoride interventions

Question 1. How effective are the different fluoride interventions in preventing dental caries in children under 5 years of age?

### Systemic fluoride

#### Collective interventions

##### Fluoridated water

The first studies reporting association between the natural fluoride content of drinking-water and decreased prevalence of caries date from 1930s. Currently, it is estimated that around 380 million people regularly consume artificially fluoridated water, in addition to 50 million who consume drinking water containing optimal fluoride concentrations naturally [4]. Recent systematic reviews have summarized the effects of the existing literature and confirmed that fluoridated water reduces the prevalence of dental caries.

One Cochrane review (Iheozor-Ejiofor 2015) evaluated the effects of water fluoridation on the prevention of dental caries [20]. The review authors included prospective controlled studies that assessed caries in a population receiving fluoridated water (naturally or artificially) and in a population receiving non-fluoridated water. The literature search was conducted up to February 2015. The review authors found very little contemporary evidence. Nine observational studies contributed to the meta-analysis on caries in deciduous teeth and were all conducted prior to 2000; six prior to 1980. The data come from Europe (five studies), Australia, Asia, North America and South America. Findings indicate that:

- The use of fluoridated water compared to low or non-fluoridated water may reduce caries in dmft, with a mean difference (MD) in the reduction of caries of 1.81 (95% confidence interval [CI] 1.31 to 2.31; 9 studies, 44,268 participants; low certainty evidence). This translates to a 35% reduction in dmft compared to the median control group mean values.
- The use of fluoridated water compared to low or non-fluoridated water may increase the percentage of caries free children by 15% in deciduous teeth (95%CI 11 to 19; 10 studies, 39,966 participants; low certainty evidence).

However, the confidence of the review authors in the size of the effect estimates ‘is limited by the observational nature of the study designs, the high risk of bias within the studies and, importantly, the applicability of the evidence to current lifestyles.’

In 2013, the US Community Preventive Services Task Force recommended fluoridation of community water sources ‘based on strong evidence of effectiveness in reducing dental caries’ [2]. These findings were based on an existing systematic review (McDonough 2000) combined with an updated search for recent evidence [15]. Most of the included studies were from the US or other high-income countries. Authors reported ‘a decrease in new dental caries after community water fluoridation began and an increase in new dental caries when it was stopped’. Their main findings showed that water fluoride initiation led to a median decrease of 15.2 percentage points in caries (12 studies), and to a decrease in caries on dmft with a median difference of − 2.25 (interquartile range − 3.63 to − 1.28; 10 studies).

This same document also reported the findings of a systematic review looking at economic evidence, stating that ‘the economic benefit of community water fluoridation is greater than the cost’ [15] (see original document for more details).

##### Fluoridated milk

‘Since 1986, the WHO International Programme for Milk Fluoridation has promoted and supported programmes aimed at demonstrating the feasibility for community use of fluoridated milk for caries prevention’ [4]. O’Mullane et al. noted the existence of three systematic reviews looking at the effects of milk fluoridation for preventing dental caries, and reported that all included studies showed a ‘reduction in dental decay among those consuming/receiving fluoridated milk’ [4]. We report below the main findings of the most recent systematic review on this topic.

One Cochrane review (Yeung 2015) assessed the effects of fluoridated milk on the prevention of dental caries at a community level [21]. Only randomized controlled trials (RCTs) comparing fluoridated versus non-fluoridated milk with an intervention and follow-up period of at least 2 years were included. The literature search was conducted up to November 2014. Only one trial was identified, conducted in Russia in 2004, unpublished. Authors judged the overall quality of the evidence as low, and found that:

- After 3 years, fluoridated milk in children compared to non-fluoridated milk may reduce caries in dmft (MD -1.14; 95%CI − 1.86 to − 0.42; one trial, 166 participants; low certainty evidence), equivalent to a prevented fraction of 31%.
- After 3 years, fluoridated milk compared to non-fluoridated milk in children may reduce caries in decayed-missing-filled teeth from the permanent dentition (MD -0.13; 95%CI − 0.24 to − 0.02; one trial, 166 participants; low certainty evidence). The disease level was very low, resulting in a small absolute effect size (not quantified).

##### Fluoridated salt

O’Mullane et al. reported that studies looking at effect of salt fluoridation for preventing dental caries found similar findings than those obtained with water fluoridation, and that ‘when most salt for human consumption is fluoridated, the community effectiveness of salt fluoridation approximates that of water fluoridation’ [4]. Studies also showed ‘acceptability by the public, no increase in individual salt consumption, no proven related increase in enamel fluorosis, no other negative health impacts reported, and very low per capita costs’.

#### Individual fluoride supplementation

One Cochrane review (Tubert-Jeannin 2011) looked at the efficacy of fluoride supplements for preventing dental caries in children [23]. Eligible criteria were RCTs or quasi-RCTs comparing fluoride supplements including tablets, drops and lozenges, with no systemic fluoride supplement or with topical fluorides or other preventive measures, with at least 2 years of follow-up. The literature search was conducted up to May 2011. Data for deciduous teeth were provided by four studies out of the 11 included studies in the review. These four studies were conducted in Sweden, Taiwan, the UK and the US, and published between 1985 and 2000. The risk of bias was judged as unclear for most of the domains for all included studies. The main findings were:

- Fluoride supplements versus no fluoride supplements: the review authors concluded that ‘the effect of fluoride supplements was unclear on deciduous or primary teeth.’ Indeed, there was a beneficial effect of fluoride supplements in children with a 73% reduction (95%CI 46 to 99) of caries in dmfs. But this was from a single small study (*n* = 175) with children with cleft lip and/or palate; certainty of the evidence was rated as very low. The effect was estimated with a 46% reduction (95%CI 8 to 83) of caries in dmft. Two studies (696 participants) were included, with high heterogeneity (no significant effect in one study, strong beneficial effect in the other study). The certainty of the evidence was rated as very low.
- Fluoride supplements versus topical fluorides or other preventive measures: there was no differential effect on deciduous tooth surfaces (13% reduction in d(m)fs; 95%CI − 7 to 33; 2 trials, 1051 participants; moderate certainty evidence).

The systematic review commissioned by the USPSTF to update their recommendations found no new trials since their previous 2004 systematic review that assessed the effectiveness of oral fluoride supplementation in children [2, 13]. Six studies were included in the 2004 systematic review. Among them, only one was a randomized trial, which is also included in the Cochrane review we described above [23]. For fluoride supplementation compared to placebo or no supplementation, authors found relative reductions from 32 to 72% for dmft, and from 38 to 81% for dmfs. The review authors concluded that ‘although the studies had some methodological limitations, such as lack of adjustment for potential confounders, inadequate blinding, or unreported attrition, and were fairly heterogeneous, they support the conclusion that oral fluoride supplementation leads to decreased dental caries in children 5 years and younger who have inadequate fluoridation in their water’ [2].

According to O’Mullane et al., ‘a number of systematic and other reviews have concluded that the quality of the reviewed studies was generally low and the evidence of a caries preventive effect on the primary and permanent dentitions was inconsistent’ [4]. After the assessment of several factors such as compliance and associated enamel fluorosis, they concluded that oral fluoride supplements have limited application as a public health measure.

##### Timing and dosage of oral fluoride supplementation

The systematic review commissioned by the USPSTF aimed to look at the evidence for timing and dosage of preventive fluoride interventions [2]. They found no studies that specifically addressed the dosage and timing of oral fluoride supplementation in children with inadequate water fluoridation. Recommendations on the dosage and on the age when to start oral fluoride supplementation are based on several factors including the level of fluoride in the water consumed by the population, and may therefore differ according to settings [2, 16].

### Topical fluoride

Topical fluorides are defined as ‘delivery systems which provide fluoride to exposed surfaces of the permanent and primary dentition, at elevated concentrations for a local protective effect and are therefore not intended for ingestion’ [4]. They can be professionally applied (gels, varnishes, foam, slow-release devices and solutions) or self-applied (toothpastes and mouth rinses).

#### Fluoride toothpastes

Fluoride toothpaste is ‘the most widely used method for maintaining a constant low level of fluoride in the oral environment’ and ‘its widespread use is considered to have played an important role in the decline in dental caries in industrialised countries in recent decades’ [4]. Several systematic reviews demonstrated the effectiveness of fluoride toothpastes in preventing dental caries [4]. We report below the findings of the two recent reviews, one published by the Cochrane Library in 2019, and one commissioned by the WHO and published in 2014 [9, 22].

One Cochrane review (Walsh 2019) looked at the effects of toothpastes of different fluoride concentrations in preventing dental caries [22]. The review authors included RCTs that compared tooth brushing with fluoride toothpaste and tooth brushing with a non-fluoride toothpaste or toothpaste of a different fluoride concentration among children between one and 6 years of age, with a minimum of one-year follow-up. The literature search was conducted up to August 2018. Eight studies were included, that were conducted in Brazil (3 studies), the UK (2 studies), China, France and Germany and published between 1982 and 2014. The main findings were:

- The use of 1500 ppm fluoride toothpaste compared to a non-fluoride toothpaste probably reduces caries in dmfs (MD of − 1.86; 95% CI − 2.51 to − 1.21; one trial, 998 participants; moderate certainty evidence).
- The use of 1450 ppm compared to 440 ppm fluoride toothpaste probably slightly reduces caries in dmft (MD -0.34; 95%CI − 0.59 to − 0.09; 1 trial, 2362 participants; moderate certainty evidence).
- The use of 1055 to 1100 ppm compared to 500 to 550 ppm fluoride toothpaste probably has similar effects in reducing caries in dmfs (MD -0.05; 95%CI − 0.38 to 0.28; two trials, 1958 participants; moderate certainty evidence), and in reducing caries in dmft (MD -0.27; 95%CI − 0.60 to 0.60; one trial, 905 participants; low certainty evidence)
- The use of 1450 ppm compared to 250 ppm fluoride toothpaste may slightly reduce caries in dmfs (MD -1.20; 95%CI − 2.92 to 0.52; 1 study, 172 participants; low certainty evidence) and in dmft (MD -0.40; 95%CI − 1.14 to 0.34; 1 study, 172 participants; low certainty evidence).

Another systematic review (Wright 2014) assessed the efficacy of fluoride toothpaste use in caries prevention among children younger than 6 years [9]. The literature search was conducted up to April 2015. Authors included all study designs other than case reports and narrative reviews. Fourteen trials that met their inclusion criteria (outcome caries) were selected, including six of the eight RCTs included in the Walsh Cochrane review. The authors judged all 14 trials with an overall high risk of bias. Their main findings were:

- Fluoride toothpaste versus control or placebo, in participants classified as ‘high-risk populations’:
  - ○ The use of 1000 to 1500 ppm toothpaste probably slightly reduces caries in dmfs (MD -0.24; 95%CI − 0.36 to − 0.13; six trials [one included in Walsh 2019]; certainty of the evidence not graded) and in dmft (MD -0.24; 95%CI − 0.41 to − 0.06; three trials [none included in Wash 2019]; certainty of the evidence not graded).
  - ○ The use of toothpaste with < 1000 ppm may slightly reduce or have similar effect on caries in dmfs (MD -0.24; 96%CI: − 0.66 to 0.18; two trials [none included in Walsh 2019]; certainty of the evidence not graded) and in dmft (MD -0.16; 95%CI − 0.46 to 0.15; two trials [none included in Walsh 2019]; certainty of the evidence not graded).
- High versus low fluoride concentration:
  - ○ The use of high (1055–1450 ppm) versus low (250–550 ppm) fluoride concentration probably has similar effects in reducing caries in dmfs (MD -0.04; 95%CI − 0.12 to 0.03; three trials [all three included in Walsh 2019]; certainty of the evidence not graded), but may slightly reduce caries in dmft (MD -0.10; 95%CI − 0.14 to − 0.05; four trials [three of them included in Walsh 2019]; certainty of the evidence not graded).

Overall, while the evidence supports the benefits of fluoride toothpastes for preventing caries in children, the evidence for the effects of the different levels of fluoride concentration is more limited. Risk of dental fluorosis for different fluoride concentrations (see below under ‘Safety of fluoride interventions’) should be considered when choosing an optimal fluoride toothpaste concentration.

#### Other topical fluoride interventions: gels and varnishes

Although mouth rinses are another topical fluoride intervention used in the prevention of dental caries, we do not review its effectiveness in this document, as we focus in children under the age of 5 years.

##### Fluoride gels

One Cochrane review (Marinho 2015) looked at the effectiveness of topically applied fluoride gels for preventing dental caries in children and adolescents [25]. The review authors included RCTs and quasi-RCTs in which the intervention was applied for at least 1 year. The literature search was conducted up to November 2014. Among the 28 included trials, three of them looked at the effectiveness of fluoride gels among children under 6 years of age for preventing dental caries in primary teeth. These three trials were conducted in Germany, the Netherlands and the US, and were published between 1978 and 2004. The two oldest trials were judged at high risk of attrition bias. Findings showed that fluoride gel versus placebo or no treatment result in a 20% reduction in dmfs (95%CI 1 to 38; three trials, 1254 participants; low certainty evidence). There were no data in dmft. We do not report findings on permanent surfaces and teeth as this is beyond the scope of this summary.

##### Fluoride varnishes

Another Cochrane review from the same first author (Marinho 2013), assessed the effectiveness of topically applied fluoride varnishes for preventing dental caries in children and adolescents [24]. Authors included RCTs and quasi-RCTs that assessed topical fluoride varnishes, using any fluoride agent at any concentration, with any technique of application and with a frequency of application of at least once a year. The literature search was up to May 2013. Twenty-two trials were included; eight were in children between one and 5 years of age for assessing caries prevention in primary teeth. However, ten trials reported data on primary teeth and were included in the meta-analysis. These ten trials were conducted in Canada (two trials), China (two trials), Sweden (two trials), Brazil, India, the UK and the US, and were published prior to 2000 (three trials) and between 2000 and 2011 (seven trials). Findings showed that fluoride varnish compared to placebo or no treatment result in a 37% reduction in d(e/m) fs (95%CI 24 to 51; 10 trials, 3804 participants; moderate certainty evidence), and in a 65% reduction in d(e/m) ft. (95%CI 48 to 82; two trials, 323; certainty of the evidence not graded). We do not report findings on permanent surfaces and teeth as this is beyond the scope of this summary.

The systematic review commissioned by the USPSTF to update their recommendations found three new trials since their previous 2004 systematic review that assessed professionally applied topical fluoride varnish in children aged 5 years and younger [14]. Two of them are included in the Cochrane review described above [24]. The third one was conducted in an Australian aboriginal community with water fluoridation levels of 0.6 ppm for more than 90% of participants, using a cluster design. It was published in 2011. They found similar findings with an association between use of fluoride varnish and decreased incidence of caries after 2 years. For the three recent studies identified, the absolute mean reductions in the number of affected tooth surfaces ranged from 1.0 to 2.4 [2, 13].

Overall, professionally-applied topical fluorides such as gels and varnishes are effective in preventing dental caries, and are generally indicated for persons at high risk of dental caries and for patients with special needs, especially in communities with low exposure to fluoride [4, 16].

##### Timing and dosage of topical fluoride interventions

No studies addressed the age at which to start and stop the use of fluoride varnish [2]. The USPSTF review authors noted that ‘available trials of fluoride varnish enrolled children ages 3 to 5 years; however, given the mechanism of action of this intervention, benefits are very likely to accrue starting at the time of primary tooth eruption.’

Regarding the effect of frequency of fluoride varnish application, ‘limited evidence found no clear effect on caries increment between performing a single fluoride varnish once every 6 months versus once a year or between a single application every 6 months versus multiple applications once a year or every 6 months’ [2]. The review authors concluded that ‘the optimum frequency of fluoride varnish application is not known.’

## Safety of fluoride interventions

Question 2. What are the potential harms of fluoride interventions for preventing caries in children under 5 years of age?

Dental fluorosis is the only scientifically proven risk of using fluoride interventions [17]. It is ‘the result of subsurface hypomineralization and porosity between the developing enamel rods’. It affects children younger than 8 years, as the permanent tooth enamel is fully mineralized in older children, except for the third molars. Both dosage and frequency of fluoride exposure during tooth development influence in the risk of fluorosis [17]. Clinically, dental fluorosis manifests from mild forms with ‘faint white lines or streaks visible only to trained examiners’ to moderate forms with mottling of the teeth (‘noticeable white lines or streaks that often coalesce into larger opaque areas’), or severe forms with ‘brown staining or pitting of the tooth enamel’ and ‘breakdown of the enamel’ [26].

### Systemic fluoride and dental fluorosis

The Cochrane review that looked at effects of fluoridated water for preventing caries, also evaluated the effects of fluoridated water on fluorosis [20]. For this outcome, 135 studies (any study design with concurrent control that compared populations exposed to different water fluoride concentrations) were included. These studies were published between 1941 and 2014, and 28% of them were conducted in Europe. All the studies were judged at high risk of bias, except four of them at unclear risk of bias, and there was substantial between-study variation. The review authors mentioned dental fluorosis as an ‘adverse effect’ for the purpose of the review, but they acknowledge that ‘moderate fluorosis may be considered an “unwanted effect” rather than an adverse effect’ and that ‘mild fluorosis may not even be considered an unwanted effect’. They report the outcomes measured as any level of dental fluorosis including mild forms, and fluorosis of aesthetic concern that refers to more severe forms. For a fluoride level of 0.7 ppm in drinking water, they found 40% of participants with any level of dental fluorosis (95%CI 35 to 44; 90 studies; 180,530 participants) and 12% of participants with fluorosis of aesthetic concern (95%CI 8 to 17; 40 studies, 59,630 participants; low certainty evidence).

The USPSTF reported adequate evidence of an association between early childhood intake of fluoride supplements and risk of fluorosis [2]. Their findings are based on a commissioned systematic review, updated with literature search up to 2006. Exposure to systemic fluoride was estimated to increase enamel fluorosis, with ORs ranging from 1.3 to 15.6 (14 observational studies based on retrospective parental recall to determine exposure to fluoride) [13]. Authors specified that fluorosis ranged ‘from mild (small white spots or streaks) to severe (discoloration, pitting, or rough enamel), depending on the overall systemic fluoride exposure level over time.’ The evidence is however limited partly due to the fact that determinations of early childhood exposures were based on retrospective parental recall [13].

After assessing the chronology of dental development and effects of systemic fluoride, Miñana et al. concluded that the excess of systemic fluoride administered before 6 years of age is an important risk factor for dental fluorosis [16].

### Topical fluoride and dental fluorosis

One Cochrane review (Wong 2010) looked at the relationship between the use of topical fluorides in children and the risk of dental fluorosis [26]. Authors included RCTs, quasi-RCTs, cohort studies, case-control studies and cross-sectional surveys that compared children under 6 years of age who received topical fluorides (toothpastes, mouth rinses, gels, foams, paint-on solutions, or varnishes) with children who received an alternative fluoride treatment, placebo or no intervention. The literature search was conducted up to March 2009 and 25 studies were included (2 RCTs, 1 cohort study, 6 case-control studies and 16 cross-sectional surveys). Studies were conducted in North-America (11 studies), Europe (nine studies), Australia (two studies), Brazil, Mexico and India. Twelve studies were conducted in non-fluoridated areas, eight in fluoridated areas, and five in both non-fluoridated and fluoridated areas. All studies except one RCT were judged to be at moderate or high risk of bias.

The systematic review conducted by Wright et al. addressed this same research question on association between use of topical fluorides and risk of dental fluorosis in children [2]. The review authors included the findings from the Wong 2010 Cochrane review and two additional studies that were published later on (one case-control study and one cross-sectional survey).

We present below the findings of different comparisons based on topical fluoride exposure from both systematic reviews. Certainty of the evidence was not graded.

- Age started using fluoride toothpaste/tooth brushing:
  - ○ From two case-control studies: reduction in risk of fluorosis in starting tooth brushing after 24 months of age compared to before 24 months of age (odds ratio [OR] 0.29; 95%CI 0.15 to 0.53), although there was high heterogeneity among the two included studies (one study showed no difference, the other showed significant difference of effect) [26]. The addition of a third case-control study to the meta-analysis decreased the magnitude of the effect (OR 0.66; 95%CI 0.48 to 0.90) [2].
  - ○ From nine cross-sectional surveys: reduction in risk of fluorosis in starting tooth brushing after 12 to 14 months of age (OR 0.70; 95%CI 0.57 to 0.88; four studies), and no difference in starting tooth brushing before and after 24 months of age (OR 0.92; 95%CI 0.71 to 1.18; five studies), with no evidence of heterogeneity [26].
- Frequency of tooth brushing: four cross-sectional surveys showed no significant association between frequency of tooth brushing and fluorosis (OR 0.88; 95%CI 0.71 to 1.08) [2, 26].
- Amount of fluoride toothpaste used: three cross-sectional surveys showed no significant association between amount of fluoride toothpaste used and fluorosis (OR 0.92; 95%CI 0.67 to 1.28) [2, 26].
- Fluoride level of toothpaste used:
  - ○ Two RCTs compared low (440 to 550 ppm) to high (1000 to 1450 ppm) fluoride level, and showed an association of low fluoride levels with a lower risk of fluorosis (relative risk [RR] 0.75; 95%CI 0.57 to 0.99 for one trial and RR 0.59; 95%CI 0.44 to 0.79 for the other trial) [26].
  - ○ Three cross-sectional surveys compared low (250 to 550 ppm) to high (≥1000 ppm) fluoride level and showed no association between fluoride level and risk of fluorosis (OR 0.79; 95%CI 0.61 to 1.02) [2, 26].

### Fluoride toxicity

The toxic dose of elemental fluoride is established at 5 to 10 mg/kg of body weight, and lethal doses at 8 to 16 mg/kg [17]. These levels are possible in children ingesting large quantities of fluoride supplements.

## Risk factors for dental caries

We list below identified risk factors for dental caries [2, 12, 16]. We do not provide an evidence-based approach to validate each factor as validated risk factors, but we provide such list from the sources identified above with the aim to provide a comprehensive document on use of fluoride interventions, as some of the interventions are indicated in children at high risk for dental caries.

### Individual factors

- Frequent sugar exposure; free sugars in foods and drinks
- Inappropriate bottle feeding; free sugars added to baby bottles
- Developmental defects of the tooth enamel (e.g. hypoplasia of enamel)
- Dry mouth (e.g. Sjögren syndrome, ectodermal dysplasia)
- Morphological alterations of the oral cavity (e.g. orofacial malformations, use of orthodontics)
- Salivary quantity (reduced flow) and constituents (particularly variations in proteins)
- Diseases that lead to high risk of dental manipulation (e.g. heart disease, immunosuppression, haemophilia and other coagulation disorders)
- History of previous caries
- Nutritional status
- Oral flora

### Maternal and family factors

- Poor oral hygiene
- Low socioeconomic status
- Recent maternal caries
- Sibling caries
- Frequent snacking
- Breastfeeding beyond 12 months, especially if frequent and/or nocturnal
- Maternal nutritional status
- Genetic susceptibility

### Other factors

- Children whose primary water supply is deficient in fluoride (defined as containing less than 0.6 ppm F)
- Lack of access to dental care
- Inadequate preventive measures (such as failure to use fluoride toothpastes)
- Lack of parental knowledge about oral health.

## Summary of findings

- Water fluoridation has been widely implemented worldwide for several decades and evidence shows it reduces the prevalence of dental caries. Salt or milk fluoridation are other collective fluoride interventions that are also effective to prevent dental caries in children.
- The evidence of effects of oral fluoride supplements for caries prevention is limited and inconsistent. By assessing several factors including compliance and dental fluorosis, the World Health Organization concluded that this intervention has limited application as a public health measure.
- The use of fluoride toothpastes has consistently been proven to be effective in the prevention of dental caries. The evidence for the effects of the different levels of fluoride concentration in toothpastes is more limited.
- The use of topical fluorides such as gels and varnishes is effective in preventing dental caries and are mainly recommended to children with high risk of dental caries.
- Early childhood intake of fluoride supplements and fluoride level of 0.7 ppm (ppm) in drinking water are associated with the risk of dental fluorosis, ranging from minor forms to severe forms that are of aesthetic concerns.
- Dental fluorosis has also been associated with the use of topical fluoride interventions. Starting tooth brushing after 12 months of age is associated with a significant reduction of dental fluorosis, but findings were inconsistent when comparing starting of tooth brushing before and after 24 months of age (data from observational studies).

## Data Availability

Not applicable.
